# Effects of *Acremonium* cellulase and heat-resistant lactic acid bacteria on lignocellulose degradation, fermentation quality, and microbial community structure of hybrid elephant grass silage in humid and hot areas

**DOI:** 10.3389/fmicb.2022.1066753

**Published:** 2022-11-21

**Authors:** Chen Chen, Yafen Xin, Xiaomei Li, Haoran Ni, Tairu Zeng, Zhaochang Du, Hao Guan, Yushan Wu, Wenyu Yang, Yimin Cai, Yanhong Yan

**Affiliations:** ^1^Department of Forage Breeding and Cultivation, College of Grassland Science and Technology, Sichuan Agricultural University, Chengdu, China; ^2^Department of Forage Efficient Conversion and Utilization, Sichuan Zoige Alpine Wetland Ecosystem National Observation and Research Station, Southwest Minzu University, Chengdu, China; ^3^Department of Crop Cultivation and Tillage, College of Agronomy, Sichuan Agricultural University, Chengdu, China; ^4^Crop, Livestock and Environmental Division, Japan International Research Center for Agricultural Sciences (JIRCAS), Tsukuba, Japan

**Keywords:** *Acremonium cellulase*, heat-resistant lactic acid bacteria, hybrid elephant grass, silage, lignocellulose degradation, microbial community

## Abstract

To better evaluate the effects of *Acremonium* cellulase (AC) and previously screened heat-resistant *Lactobacillus plantarum* 149 (LP149) on lignocellulose degradation, fermentation quality, and microbial community during ensiling in humid and hot areas, this study used a small-scale fermentation system to prepare hybrid elephant grass silage at 30 and 45°C, respectively. Compared to control and commercial inoculant *Lactobacillus plantarum* (LP), the addition of AC or strain LP149 decreased the contents of neutral detergent fiber, acid detergent fiber, and cellulose and increased the contents of glucose, fructose, and sucrose during fermentation. Furthermore, AC and LP149 treatments altered the microbial communities' structure during ensiling. AC treatment provided more substrate for microbial fermentation, resulting in an increase in bacterial alpha diversity. LP149 treatment increased the *Lactobacillus* abundance and optimized the bacterial community compositions. In addition, AC and LP149 treatments had higher (*P* < 0.05) lactic acid and acetic acid contents and lower (*P* < 0.05) pH, butyric acid, and NH_3_-N levels compared to the control. These results indicated that AC and strain LP149 are promising silage additives that can promote lignocellulose degradation and improve the fermentation quality of hybrid elephant grass in humid and hot areas.

## Introduction

Energy is the material foundation for human survival and development. In recent years, the increasing global energy crisis and the ecological environmental issue caused by traditional energy have brought biomass energy to a higher status. Biomass energy is a renewable and clean energy source that not only effectively reduces carbon emissions but also improves agricultural yields and efficiency (Liu et al., 2021). Elephant grass is one of the most promising energy plants that is widely used as feed in humid and hot areas because of its high yield and powerful regeneration capacity, and it also has many biomass-related quality attributes (Strezov et al., 2008; Araújo et al., 2017). Furthermore, the stalks of elephant grass are rich in lignocellulose, which is the world's most abundant polymeric carbohydrate and an important raw material to produce fermentable sugars (Hosseini Koupaie et al., 2019). However, lignocellulose is a complex and stubborn substance that is difficult to break down into desirable products (Prasad et al., 2019). Anaerobic fermentation is an effective pretreatment method to convert lignocellulose to energy products (Hosseini Koupaie et al., 2019).

Ensiling is a promising technology that uses lactic acid bacteria (LAB) for anaerobic fermentation to extend the storage time of feeds through acidification (Guan et al., 2018). Ensilage preserves more than 90% of plant energy and is an important pretreatment method for producing energy products (Zhao et al., 2017; Li et al., 2019). However, conventional ensiling technology in humid and hot areas faces great challenges. The optimum temperature of LAB is 20–30°C. Too high or low temperatures are not suitable for the LAB to grow (Zhou et al., 2016). But in the initial stage of fermentation, the raw materials use the residual air in the silos for respiration and release heat, which can result in temperatures of up to 40°C or higher (Li et al., 2019), particularly in humid and hot areas, with high temperature and rainfall, which can easily lead to poor fermentation quality.

Exogenous addition is a key measure to promote lignocellulose degradation and improve fermentation quality. The additives commonly used are LAB and enzyme preparations. Studies reported that commercial inoculant LAB has no desirable effect in humid and hot areas, and strains isolated from local silage materials are effective in improving the silage quality (Santos et al., 2013; Pholsen et al., 2016). Heat-resistant LAB, screened from silage in humid and hot areas, has the potential to cope with global warming and improve the fermentation quality in this region (Guan et al., 2020). Yet, the effect of heat-resistant LAB on the silage quality of high-moisture hybrid elephant grass is not clear, and less information is available on lignocellulose degradation. *Acremonium* cellulase can directly convert part of lignocellulose into soluble sugar to provide substrate for microbial fermentation under normal conditions (Li et al., 2018a). But it needs to be further investigated whether *Acremonium* cellulase has a desirable effect in humid and hot areas, and its effectiveness in improving fermentation quality is controversial.

From the above, there is an importance for developing silage additives that can promote lignocellulose degradation and improve fermentation quality in humid and hot areas. The characteristics of *Acremonium* cellulase and heat-resistant LAB reflect their potential as silage additives for utilization in humid and hot areas. Therefore, this study was performed to evaluate the effects of *Acremonium* cellulase and heat-resistant LAB on lignocellulose degradation, fermentation quality, and microbial communities of hybrid elephant grass in humid and hot areas. The findings might provide useful information for the development of high-quality silage additives in humid and hot areas.

## Materials and methods

### Materials and silage additives

The hybrid elephant grass [(*Pennisetum americanum* × *Pennisetum purpureum*) × *Pennisetum durpureum schum*. cv. Guimu No. 1] was harvested from the Chongzhou experiment farm of Sichuan Agricultural University (N30°33′23.98″ E103°38′42.61″) on August 26, 2020, at which time the hybrid elephant grass was at nutritional growth period (1.8–2.0 m). These materials were chopped to 2–3 cm for the preparation of silage. The silage additives include heat-resistant *Lactobacillus plantarum* 149 (LP149), commercial inoculant *Lactobacillus plantarum* (LP, Sichuan Gaofuji Biotech Co., Ltd, Chengdu, China), and *Acremonium* cellulase (AC, Green Stone Swiss Co., Ltd., Shanghai, China). Strain LP149 was screened previously from silages in Southwest China as a humid and hot area, which has good growth characteristics that tolerate the low pH (3.5) and high temperature (45°C). The gene sequence of this strain has been uploaded to GenBank with the registration number MH 337263. LP and AC were supplied as lyophilized powders, where LP has a viable count of 50 billion colony-forming units (cfu)/g and AC has an enzymatic activity of over 1,000 U/g.

### Ensiling of hybrid elephant grass

The chopped hybrid elephant grass was mixed thoroughly with three additives separately, packed into polyethylene plastic bags (200 × 300 mm, Shenzhen Sanfeng Plastic Packing Co., Ltd., Shenzhen, China, each bag weighed 300 ± 2 g) and sealed with vacuum sealer (DJVac, Wenzhou, China). AC was applied at a rate of 0.03% FW. LP and LP149 were inoculated at a rate of 5 × 10^6^ cfu/g FW. The appropriate amounts of AC and LP lyophilized powder were filled into the sterilized small spray and diluted with 10 mL of sterile water. An equal volume of sterile water was added to the hybrid elephant grass as a control group. Strain LP149 requires activation in MRS (De Man, Rogosa, and Sharpe) broth medium before inoculation and detection of viable bacteria by plate count method (Xie et al., 2022). In this study, a total of 120 bags of silage samples were prepared (4 treatments × 2 ensiling temperatures × 5 ensiling times × 3 replicates). The plastic bag silos were stored in incubators at 30 or 40°C for 1, 3, 7, 14, and 60 days of ensiling.

### Sampling and analytical method

The plastic bag silos were opened at the designed ensiling time to analyze the fermentation and chemical characteristics. A 20 g sample of hybrid elephant grass silage was placed in a juicer, then 180 mL of distilled water was added and blended at high speed for 45 s, and the residue was removed through 4 layers of gauze. The obtained filtrate was poured into a clean conical flask, and the pH value was measured immediately. A volume of 50 mL of the filtrate, preserved in a freezer at −20°C, was used for subsequent analysis. The ammonia nitrogen (NH_3_-N) content was analyzed by the phenol-hypochlorite method (Li et al., 2021). The organic acids were detected using a high-performance liquid chromatograph according to the procedure of Yan et al. (2019).

About 100 g of fresh hybrid elephant grass and silage samples were placed in a blast dryer at 65°C and dried for 3 days to determine the dry matter (DM) content. An appropriate amount of dried samples was crushed, sieved (1 mm), and stored in a desiccator for chemical composition analysis. The water-soluble carbohydrate (WSC) was detected using the anthrone-sulfuric acid method (AOAC, 1990). The nitrogen was analyzed by the Dumas combustion method (Rapid N exceed, Elementar, Germany), and a protein conversion coefficient (6.25) was used to calculate crude protein. The neutral detergent fiber (NDF), acid detergent fiber (ADF), and acid detergent lignin (ADL) were determined according to the method of Van Soest et al. (1991). Hemicellulose and cellulose contents were estimated from the differences between NDF and ADF, ADF and ADL contents, respectively (Li et al., 2019). The sucrose, glucose, and fructose contents were detected with a kit (Hexokinase method, G0545W, Suzhou Grace Biotechnology Co., Ltd, Suzhou, China).

### Bacterial community analysis

#### High-throughput sequencing

The genomic DNA from the hybrid elephant grass silage was extracted using the bacterial DNA isolation kit (DE-05311, Foregene, Chengdu, China). DNA concentration and purity were detected by NanoDrop2000C, with the optical density set at 260/280 nm (Yan et al., 2019). Qualified DNA samples were used for subsequent analysis. The 16S rRNA genes of distinct regions (V3–V4) were amplified by PCR with the primers 341F (5'-CCTAYGGGRBGCASCAG-3') and 806R (5'-GGACTACNNGGGTATCTAAT-3'). Qualified PCR products were selected using agarose gel electrophoresis at 2% concentration, and then the target bands were recovered by the gel extraction kit (Qiagen, Germany). Finally, libraries were constructed, and qualified libraries were sequenced on the platform of Illumina NovaSeq6000.

#### Sequences analysis

The raw sequencing data were spliced and filtered to select high-quality tags and were compared with the database of species annotation to remove chimeric sequences, and hence effective tags were obtained (Haas et al., 2011). Based on 97% of identification, the sequences of these tags were clustered into OTUs according to the Uparse algorithm (version 7.0.1001). The representative sequences from the OTUs were selected for species annotation (Wang et al., 2007). The bacterial community compositions of all samples were analyzed. Then, the alpha diversity index of hybrid elephant grass silages was calculated using the Qiime software (version 1.9.1). The R software (version 4.1.3) was used for the principal coordinate analysis (PCoA) of bacterial communities at different ensiling days.

### Statistical analyses

One-way ANOVA and multi-way ANOVA were performed using SPSS (version 24.0) for the additives, ensiling time, and temperature. Duncan's new multiple-range test was used to compare the mean values of different chemical compositions and fermentation characteristics. Plate count results of microorganisms need to be log-transformed before statistical analysis. Differences were regarded as statistically significant when *P* < 0.05.

## Results and discussion

### Chemical characteristics and microbial population of fresh hybrid elephant grass

The initial DM content of fresh hybrid elephant grass was 172.62 g/kg DM. It is difficult to reduce the moisture content of fresh grass by wilting in humid and hot areas. High-moisture grass easily leads to undesirable microbial fermentation, nutrient loss, and aerobic spoilage during ensiling. In addition, the WSC, CP, NDF, ADF, and ADL contents of fresh hybrid elephant grass were 76.51, 103.82, 584.16, 336.28, and 33.73 g/kg DM, respectively. The number of LAB, coliform bacteria, yeast, and mold was 2.03, 4.00, 2.49, and 1.75 log_10_ cfu/g FM, respectively. It is generally believed that the number of LAB ≥ 10^5^ cfu/g FM is more preferable for the preservation of silage (Ni et al., 2017). Therefore, exogenous addition is a key measure to improve the fermentation quality of high-moisture hybrid elephant grass in humid and hot areas.

### Fermentation characteristics of hybrid elephant grass silage

The fermentation characteristics of hybrid elephant grass silages treated with additives at different ensiling times and temperatures are presented in Table 1. Compared to the control, three additive groups had a lower pH and were ≤ 4.2 after 3 days of ensiling, which is the threshold value indicating that the silages were well preserved (Mu et al., 2020). In addition, the pH decline rate after 1 day of ensiling at two temperatures was LP > LP149 > AC. The pH decline rate is regarded as a more important indicator for reflecting fermentation dynamics than the final pH value (Mu et al., 2020). However, it does not mean that lower pH is better, as the growth of LAB is suppressed when the pH ≤ 3.8 (Muck, 2010). The pH of AC and LP treatments decreased below 3.8 after 7 days of ensiling at 30°C. The accumulation of organic acids is the main reason for the pH decrease during anaerobic fermentation (Wang et al., 2021). Thus, AC and LP treatments may have facilitated the production of organic acids. Interestingly, the pH of all silages slightly increased after 60 days of ensiling at 45°C. With the soluble sugars depleted in the late stage of high-temperature fermentation, some acid-tolerant undesirable microorganisms such as yeasts take organic acids as a carbon source to proliferate, leading to a rise in pH.

**Table 1:** Fermentation characteristics of hybrid elephant grass silages treated with additives at different ensiling times and temperatures.

**Items**	**Additive**	**30°**C					**45°**C					**SEM**	***P*** **-value**						
		**1 days**	**3 days**	**7 days**	**14 days**	**60 days**	**1 days**	**3 days**	**7 days**	**14 days**	**60 days**		**T**	**A**	**D**	**T × A**	**T × D**	**A × D**	**T × A × D**
pH	C	4.77^b^	4.25^b^	4.00^c^	3.95^d^	3.92^d^	5.04^a^	4.29^a^	4.19^a^	4.32^a^	4.33^a^	0.024	**	**	**	**	**	**	**
	AC	4.32^c^	4.00^f^	3.78^e^	3.78^f^	3.70^f^	4.29^c^	4.15^d^	4.09^b^	4.03^c^	4.20^b^								
	LP	4.07^e^	3.80^g^	3.76^e^	3.71^g^	3.78^e^	4.20^d^	4.09^e^	3.99^c^	4.06^c^	4.10^c^								
	LP149	4.21^d^	4.19^c^	3.93^d^	3.91^e^	3.86^d^	4.23^d^	4.20^c^	4.18^a^	4.14^b^	4.18^b^								
Lactic acid (g/kg DM)	C	30.74^de^	39.47^f^	82.64^c^	85.45^c^	91.98^c^	29.07^e^	32.54^h^	40.67^f^	49.32^e^	47.56^f^	2.537	**	**	**	**	**	**	**
	AC	31.57^d^	55.94^c^	86.81^b^	107.52^b^	121.84^a^	31.86^d^	35.14^g^	42.85^ef^	50.08^e^	51.35^e^								
	LP	56.45^a^	88.07^a^	91.64^a^	116.09^a^	108.26^b^	44.15^b^	48.78^d^	52.31^d^	62.59^d^	59.93^d^								
	LP149	36.54^c^	61.19^b^	85.10^b^	104.37^b^	119.46^a^	37.85^c^	43.17^e^	45.09^e^	50.78^e^	53.07^e^								
Acetic acid (g/kg DM)	C	8.83^cd^	10.69^c^	13.60^c^	15.88^d^	16.53^b^	8.96^cd^	10.72^c^	12.73^c^	13.59^e^	16.08^b^	0.426	ns	**	**	**	*	**	ns
	AC	10.38^bc^	14.84^b^	16.15^b^	19.13^bc^	21.97^a^	13.56^a^	18.07^a^	18.52^ab^	18.34^c^	20.93^a^								
	LP	7.52^d^	9.72^c^	9.59^d^	10.16^f^	12.63^c^	8.34^cd^	9.30^c^	9.72^d^	9.78^f^	12.01^c^								
	LP149	13.28^a^	19.03^a^	19.80^a^	21.70^a^	22.40^a^	10.55^b^	18.74^a^	19.64^a^	20.17^ab^	21.58^a^								
Propionic acid (g/kg DM)	C	ND	0.60^ab^	0.78^b^	1.06^a^	1.24^a^	ND	0.51^ab^	0.60^c^	0.72^bc^	0.77^e^	0.037	ns	**	**	**	**	**	*
	AC	ND	ND	0.51^c^	0.62^c^	0.98^cd^	ND	ND	0.59^c^	0.74^bc^	0.89^de^								
	LP	ND	0.46^b^	0.62^c^	0.76^bc^	1.13^bc^	ND	0.54^ab^	0.87^ab^	0.91^ab^	1.08^bc^								
	LP149	ND	0.62^a^	0.78^b^	1.01^a^	1.25^a^	ND	0.56^ab^	0.96^a^	1.03^a^	1.06^c^								
Butyric acid (g/kg DM)	C	ND	0.65^b^	0.87^a^	1.38^c^	2.78^c^	ND	0.94^a^	1.13^a^	1.67^b^	3.49^a^	0.084	**	**	**	**	**	**	**
	AC	ND	ND	0.42^b^	0.81^de^	1.63^f^	ND	ND	0.38^b^	0.84^de^	1.70^ef^								
	LP	ND	0.51^b^	0.93^a^	1.69^ab^	2.06^d^	ND	0.83^a^	1.06^a^	1.85^a^	3.26^b^								
	LP149	ND	ND	ND	0.76^e^	1.57^f^	ND	ND	0.62^b^	0.98^d^	1.91^de^								
NH_3_-N (g/kg TN)	C	6.32^cd^	14.08^c^	26.14^d^	56.41^a^	70.62^bc^	9.15^a^	20.23^a^	39.41^a^	57.52^a^	75.64^a^	2.121	**	**	**	*	**	**	ns
	AC	4.91^de^	10.41^d^	23.67^de^	48.28^c^	65.36^d^	8.83^ab^	17.06^b^	35.32^b^	52.36^b^	71.85^b^								
	LP	4.17^e^	8.95^de^	25.46^de^	40.79^d^	64.23^d^	7.53^bc^	14.59^c^	32.18^c^	43.65^d^	67.42^cd^								
	LP149	3.84^e^	8.06^e^	22.78^e^	43.54^d^	60.57^e^	7.96^ab^	15.37^bc^	30.95^c^	47.04^c^	69.18^bc^								

a Values with different lowercase letters show significant differences among treatments on the same ensiling day (P < 0.05); DM, dry matter; ND, not detected; SEM, standard error of means; ns, not significant; ^*^P < 0.05; ^**^P < 0.01.

b C, control; AC, *Acremonium* cellulase; LP, commercial inoculant *Lactobacillus plantarum*; LP149, *Lactobacillus plantarum* 149.

c T, ensiling temperature; A, additive; D, ensiling time (d); T × A, the interaction between ensiling temperature and additive; T × D, the interaction between ensiling temperature and time; A × D, the interaction between additive and ensiling time; T × A × D, the interaction between ensiling temperature, additive and ensiling time.

Lactic acid (LA) is the most powerful organic acid capable of rapidly decreasing pH (Ali et al., 2020). Compared to the control, the silages treated with additives increased the LA content. The LP treatment had a higher (*P* < 0.05) LA content than the other treatments in the first 14 days of ensiling, but it decreased to different degrees at two temperatures after 60 days of ensiling. It is reported that some undesirable microorganisms can use LA as a substrate for fermentation when the soluble carbohydrate is low in late fermentation (Oliveira et al., 2017). The AC and LP149 treatments had higher LA content than the control during the whole fermentation process and were higher (*P* < 0.05) than the LP treatments after 60 days of ensiling at 30°C. These suggest that the addition of AC or strain LP149 may be more preferable than LP for the long-term preservation of silage.

The appropriate amount of acetic acid (AA) can enhance the aerobic stability of the silage to a certain extent (Kleinschmit and Kung, 2006). Throughout the fermentation process, AC and LP149 treatments had a higher (*P* < 0.05) AA content than the control and LP treatments. AC treatment can lead to the hydrolysis of lignocellulose to pentose, thus promoting the production of AA (Li et al., 2018b). The strain LP149 may boost the production of metabolites with bacteriostatic activity during ensiling. The LA/AA value is usually considered a qualitative indicator of ensiling, with a good fermentation ratio of about 2.5–3.0 (Guan et al., 2020). After 60 days of ensiling, the LA/AA of LP149 treatment at two temperatures (5.33 and 2.52) was closest to the ideal ratio, followed by AC treatment (5.55 and 2.45), while the LP treatment had a higher ratio (8.57 and 4.99). This may indicate that AC and LP149 are more preferable than LP for enhancing aerobic stability of silage in humid and hot areas. Throughout the ensiling process, a slight amount of propionic acid and butyric acid was detected (< 3.5 g/kg DM). It was worth noting that butyric acid content was lower (*P* < 0.05) in AC and LP149 treatments than that in the control and LP treatments.

A high concentration of NH_3_-N is the result of excessive protein degradation. In this study, the NH_3_-N concentration in all silages (< 75 g/kg TN) was consistent with the criteria for good quality silage (< 100 g/kg TN) (Mu et al., 2020). After 14 days of ensiling, the three additive groups had lower (*P* < 0.05) NH_3_-N levels than the control. This might be because the additive treatments rapidly lowered the pH, which inhibits the undesirable bacteria from degrading the protein. Moreover, the silages stored at 45°C had higher NH_3_-N levels than those stored at 30°C. It was reported that high temperature usually leads to butyric acid fermentation and more protein breakdown during ensiling (Chen et al., 2013).

### Variations in lignocellulose compositions during ensiling

The lignocellulose compositions (including NDF, ADF, ADL, cellulose, and hemicellulose) of hybrid elephant grass silages treated with additives at different ensiling times and temperatures are presented in Table 2. The AC treatment had lower (*P* < 0.05) contents of NDF, ADF, and cellulose than the other treatments after 7 days of ensiling. This reflected the powerful potential of AC to the degradation of lignocellulose during ensiling in humid and hot areas. Moreover, the LP149 treatment had lower (*P* < 0.05) contents of NDF, ADF, and cellulose than the LP treatment after 14 days of ensiling. This may prove that strain LP149 has better heat resistance and cellulolytic activity than LP.

**Table 2:** Lignocellulose compositions of hybrid elephant grass silages treated with additives at different ensiling times and temperatures.

**Items**	**Additive**	**30°**C					**45°**C					**SEM**	***P*** **-value**						
		**1 days**	**3 days**	**7 days**	**14 days**	**60 days**	**1 days**	**3 days**	**7 days**	**14 days**	**60 days**		**T**	**A**	**D**	**T × A**	**T × D**	**A × D**	**T × A × D**
NDF (g/kg DM)	C	585.34	579.53^a^	573.80^a^	568.86^a^	561.17^a^	579.72	573.54^bc^	567.53^bc^	561.39^bc^	547.57^bc^	2.079	ns	**	**	**	**	**	**
	AC	577.51	570.0^c^	551.63^e^	530.34^f^	504.65^f^	576.18	564.87^d^	538.97^f^	521.62^g^	483.37^g^								
	LP	581.33	573.23^bc^	565.54^cd^	548.07^d^	539.89^d^	577.56	575.68^ab^	570.85^ab^	566.05^ab^	551.93^b^								
	LP149	579.57	572.03^bc^	561.41^d^	537.03^e^	521.52^e^	582.45	577.16^ab^	566.58^bc^	558.96^c^	543.84^cd^								
ADF (g/kg DM)	C	340.88	339.08^a^	335.02^a^	333.58^a^	327.59^a^	338.03	333.55^b^	328.76^bc^	325.04^b^	316.03^b^	1.679	ns	**	**	**	**	**	**
	AC	337.77	331.78^b^	315.33^e^	297.13^f^	279.81^e^	339.89	330.45^b^	306.27^f^	292.51^f^	257.14^f^								
	LP	335.62	329.43^b^	323.35^cd^	309.59^d^	304.39^c^	334.35	332.12^b^	329.41^b^	327.30^b^	319.71^b^								
	LP149	336.10	330.87^b^	319.36^de^	302.72^e^	292.08^d^	335.37	332.96^b^	323.14^cd^	317.15^c^	304.58^c^								
ADL (g/kg DM)	C	35.53	36.08	35.20	35.84	35.17	36.26	34.53	35.22	34.35	34.13	0.150	ns	ns	ns	ns	ns	ns	ns
	AC	36.03	35.15	34.64	35.36	34.85	35.64	34.84	34.27	34.54	34.28								
	LP	36.85	35.22	36.26	35.42	34.39	35.75	35.26	34.43	34.29	34.56								
	LP149	35.71	35.40	36.33	33.70	33.54	36.04	35.15	34.97	36.08	35.27								
Cellulose (g/kg DM)	C	305.34	302.99^a^	299.82^a^	297.74^a^	292.42^a^	301.77	299.02^ab^	293.54^bc^	290.68^b^	281.90^b^	1.649	ns	**	**	**	*	**	**
	AC	301.74	296.63^b^	280.69^e^	261.78^e^	244.96^e^	304.25	295.60^b^	272.00^f^	257.98^e^	222.86^f^								
	LP	298.77	294.22^b^	287.09^d^	274.17^d^	270.01^c^	298.59	296.86^b^	294.98^ab^	293.01^ab^	285.15^b^								
	LP149	300.40	295.47^b^	283.03^de^	269.03^d^	258.54^d^	299.34	297.81^ab^	288.17^cd^	281.07^c^	269.32^c^								
Hemicellulose (g/kg DM)	C	244.47	240.46^ab^	238.78^ab^	235.28^bc^	233.58^ab^	241.69	239.99^ab^	238.77^ab^	236.35^ab^	231.54^ab^	0.619	ns	**	**	*	ns	ns	ns
	AC	239.74	238.23^ab^	236.30^ab^	233.21^bc^	224.84^b^	236.29	234.42^b^	232.70^b^	229.11^c^	226.23^b^								
	LP	245.71	243.80^a^	242.19^ab^	238.48^ab^	235.50^ab^	243.22	243.56^a^	241.45^ab^	238.75^ab^	232.22^ab^								
	LP149	243.47	241.16^a^	242.05^ab^	234.31^bc^	229.44^ab^	247.08	244.21^a^	243.44^a^	241.81^a^	239.26^a^								

a Values with different lowercase letters show significant differences among treatments on the same ensiling day (P < 0.05); DM, dry matter; NDF, neutral detergent fiber; ADF, acid detergent fiber; ADL, acid detergent lignin; SEM, standard error of means; ns, not significant; ^*^P < 0.05; ^**^P < 0.01.

b C, control; AC, *Acremonium* cellulase; LP, commercial inoculant *Lactobacillus plantarum*; LP149, *Lactobacillus plantarum* 149.

c T, ensiling temperature; A, additive; D, ensiling time (d); T × A, the interaction between ensiling temperature and additive; T × D, the interaction between ensiling temperature and time; A × D, the interaction between additive and ensiling time; T × A × D, the interaction between ensiling temperature, additive and ensiling time.

After 3 days of ensiling, the LP and LP149 silages stored at 45°C had a higher content of NDF, ADF, and cellulose than those stored at 30°C. Generally, high temperatures can result in the deposition of lignocellulose in silage (Wilson et al., 1991). The Maillard reaction is promoted when the ensiling temperature is higher than 35–40°C, and the polymer produced by this reaction increases the lignocellulose in silage (Muck et al., 2003). However, the control and AC silages stored at 45°C had a lower content of NDF, ADF, and cellulose than those stored at 30°C. This is probably due to the high temperature promoting the growth of epiphytic bacteria with cellulolytic activity, and the optimum temperature of AC is 50–60°C (Li et al., 2018a).

### Variations in fermentable carbohydrates contents during ensiling

**Figure 1:**
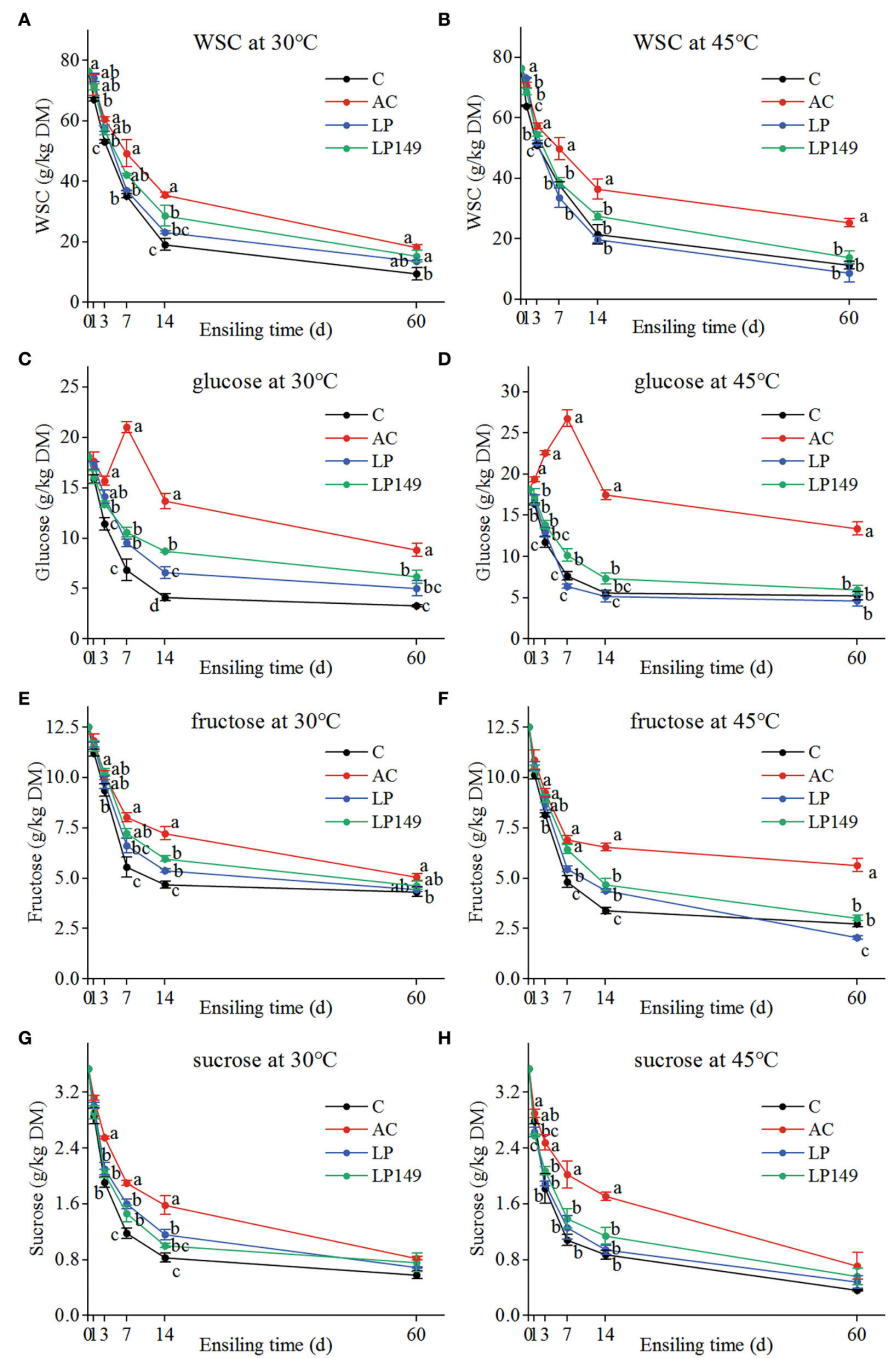
Variations in the contents of water-soluble carbohydrates (WSC) **(A,B)**, glucose **(C,D)**, fructose **(E,F)**, and sucrose **(G,H)** of hybrid elephant grass silages treated with additives at different ensiling times and temperatures. C, control; AC, *Acremonium* cellulase; LP, commercial inoculant *Lactobacillus plantarum*; LP149, *Lactobacillus plantarum* 149. Means with different small letters show the significant difference among treatments in the same ensiling days at *P* < 0.05 (*n* = 3, bars indicate standard error of means).

The variations in the contents of fermentable carbohydrates (WSC, glucose, fructose, and sucrose) of hybrid elephant grass silages treated with additives at different ensiling times and temperatures are shown in Figure 1. Compared to the control, the contents of fermentable carbohydrates in three additive groups were higher (except for the LP treatment at 45°C). Throughout the fermentation process, apart from the glucose content of the AC treatment that appeared an increasing trend in the first 7 days of ensiling, other fermentable carbohydrates decreased gradually with the extension of ensiling time, which decreased sharply in the first 7 or 14 days of ensiling and then decreased slowly. This is closely related to the anaerobic fermentation process, where a large amount of fermentable sugar is consumed during the aerobic respiration period and the intensive fermentation phase, but the demand for substrates decreases when fermentation enters a stable phase (Ávila and Carvalho, 2020). In addition, the increased glucose content of the AC treatment in the first 7 days of ensiling may be attributed to the accumulation of glucose as a result of AC promoting the degradation of lignocellulose to produce more glucose than that consumed by microbial fermentation (Li et al., 2019). All fermentable carbohydrates of LP149 treatment were higher than LP treatment after 7 days of ensiling (except for sucrose at 30°C). This may indicate that strain LP149 was more beneficial than LP in promoting the degradation of lignocellulose during ensiling of hybrid elephant grass.

**Figure 2:**
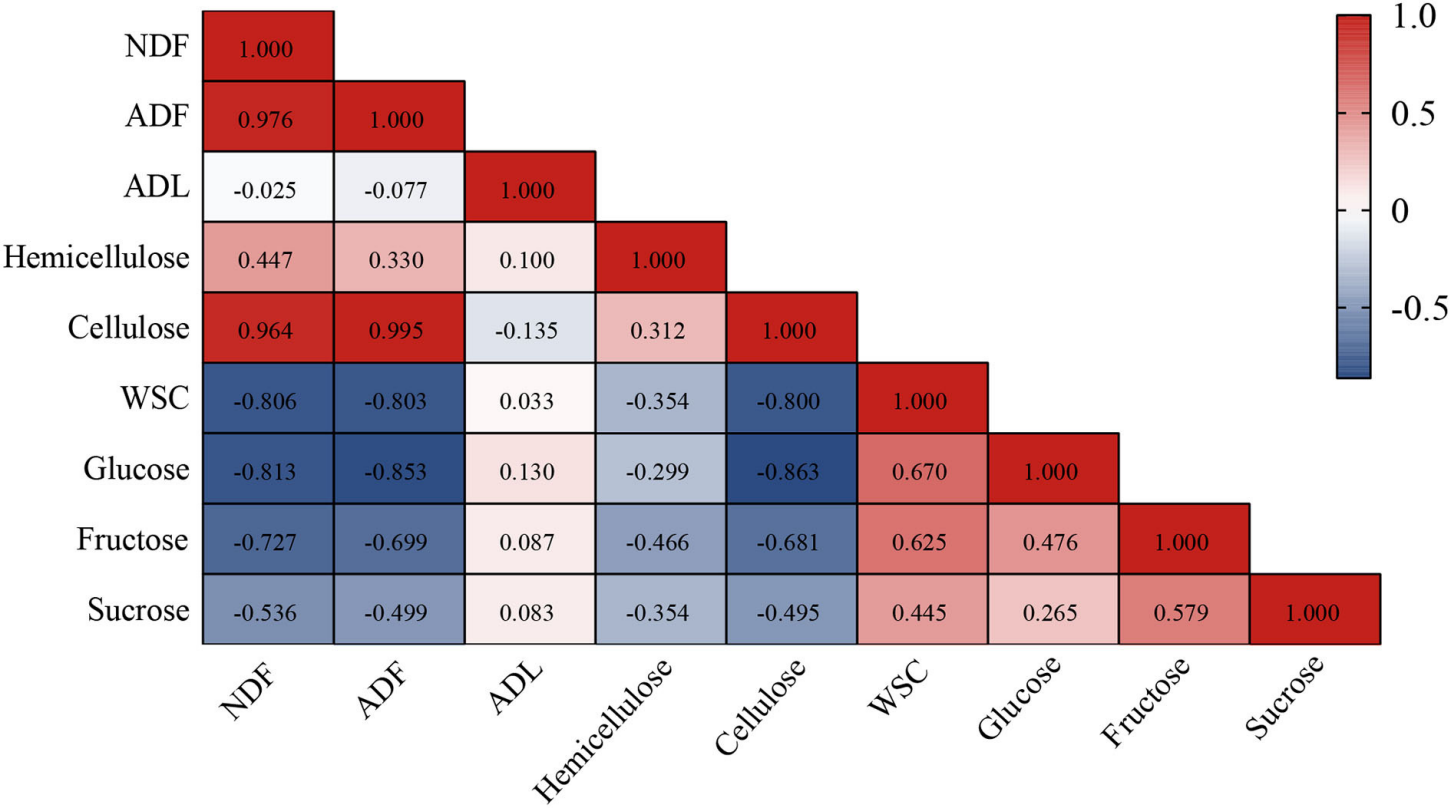
The Spearman's correlation analysis between lignocellulose (including NDF, ADF, ADL, hemicellulose, and cellulose) and fermentable carbohydrates (including WSC, glucose, fructose, and sucrose) of hybrid elephant grass ensiled at 60 days. NDF, neutral detergent fiber; ADF, acid detergent fiber; ADL, acid detergent lignin; WSC, water-soluble carbohydrates.

Correlation analysis of the compositions of lignocellulose and fermentable carbohydrates was performed to explore the linkage between them (Figure 2). Overall, lignocellulose was negatively correlated with fermentable carbohydrates where NDF, ADF, and cellulose were negatively (*P* < 0.01) correlated with WSC, glucose, and fructose. Taking into account the variation of their contents, this may demonstrate that lignocellulose is mainly degraded to WSC, glucose, and fructose during ensiling, whereas WSC mainly consists of glucose, fructose, and sucrose (Usman et al., 2022).

### Bacterial diversity during ensiling

The Goods coverage of all silages was >99%, providing a possibility of microbial communities analysis (Table 3). In conventional silage, LAB inoculation could reduce alpha diversity (Bai et al., 2021; Liu et al., 2022). But in this study, three additive groups did not have the desired effect to reduce alpha diversity, and even alpha diversity was higher than the control. This is probably due to that the humid and hot environments promoted the growth of undesirable microorganisms, and the additive treatments indirectly provided them with fermentation substrates (Zhao et al., 2019).

**Table 3:** Alpha diversity of hybrid elephant grass silages treated with additives at different ensiling times and temperatures.

**Item**	**30°**C						**45°**C				
	**Goods_coverage**	**Chao1**	**Shannon**	**Simpson**	**ACE**		**Goods_coverage**	**Chao1**	**Shannon**	**Simpson**	**ACE**
C-1	1.000	147.51	3.47	0.86	155.55		0.999	169.40	3.14	0.83	176.75
AC-1	1.000	143.13	2.86	0.77	148.85		0.999	167.25	3.18	0.83	172.95
LP-1	0.999	194.68	2.56	0.74	201.68		1.000	157.23	3.20	0.83	157.53
LP149-1	0.999	181.94	3.59	0.87	191.74		0.999	192.04	3.43	0.84	198.96
C-3	1.000	147.22	3.25	0.82	149.07		1.000	158.19	3.38	0.83	161.53
AC-3	1.000	148.92	3.52	0.85	154.48		0.999	174.38	3.54	0.84	180.10
LP-3	1.000	155.91	3.31	0.82	159.81		1.000	158.97	3.40	0.84	162.25
LP149-3	1.000	156.86	2.88	0.72	165.36		0.999	176.47	3.19	0.79	182.18
C-7	0.999	231.40	3.66	0.86	239.34		0.999	254.09	3.56	0.84	259.09
AC-7	0.999	321.15	4.01	0.90	329.91		0.999	376.63	4.23	0.91	387.60
LP-7	0.999	258.72	3.53	0.82	263.82		0.999	351.19	3.92	0.88	361.06
LP149-7	0.999	268.76	3.57	0.85	272.14		0.999	303.62	3.88	0.88	309.53
C-14	0.998	497.64	3.79	0.85	510.13		0.997	488.39	3.34	0.80	527.45
AC-14	0.997	553.62	4.14	0.90	573.47		0.998	518.20	4.05	0.86	524.91
LP-14	0.998	338.89	2.97	0.71	353.86		0.999	532.97	4.12	0.89	536.14
LP149-14	0.999	349.84	3.27	0.79	365.04		0.998	530.69	4.36	0.91	542.03
C-60	0.999	424.28	3.93	0.86	433.45		0.999	248.86	1.34	0.30	255.65
AC-60	0.998	503.62	4.24	0.90	520.88		0.999	441.02	5.13	0.92	441.03
LP-60	0.998	462.71	3.71	0.83	474.77		0.999	500.62	5.10	0.91	505.22
LP149-60	0.997	453.88	3.48	0.78	477.43		0.998	451.69	5.16	0.93	464.31

C, control; AC, *Acremonium* cellulase; LP, commercial inoculant *Lactobacillus plantarum*; LP149, *Lactobacillus plantarum* 149. The number after the treatment zone represents the ensiling time: 1, 3, 7, 14, and 60 represent 1, 3, 7, 14, and 60 days of ensiling, respectively.

**Figure 3:**
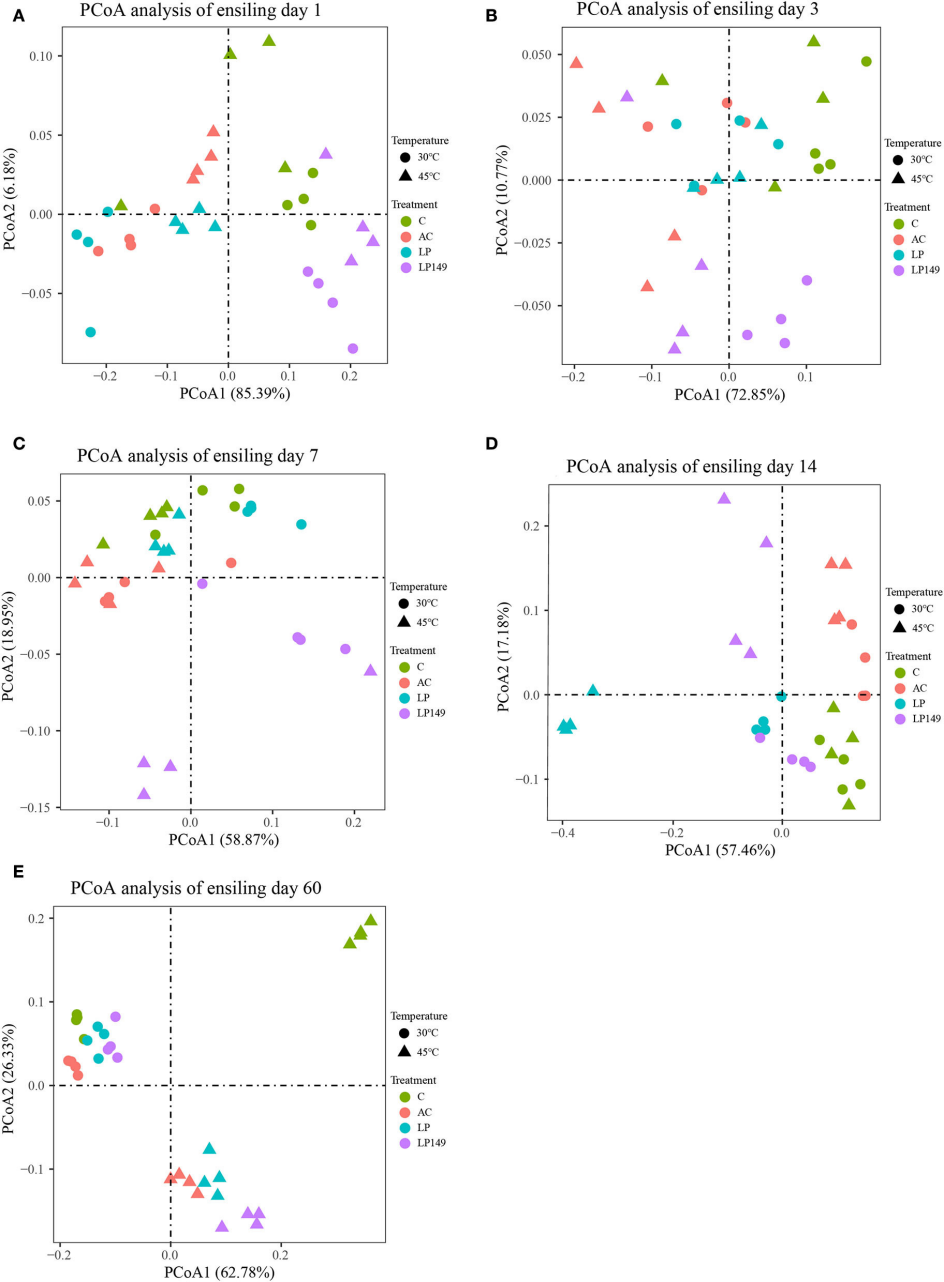
Principal coordinates analysis (PCoA) of the bacterial community of hybrid elephant grass during ensiling. **(A–E)** indicates PCoA analysis at ensiling day 1, 3, 7, 14, and 60, respectively. C, control; AC, *Acremonium* cellulase; LP, commercial inoculant *Lactobacillus plantarum*; LP149, *Lactobacillus plantarum* 149.

PCoA reflected the similarity or dissimilarity of bacterial community composition during ensiling (Figure 3). Based on PCoA at different ensiling times (Figures 3A–E), bacterial communities were clearly separated among control and treated groups after 1, 3, 7, 14, and 60 days of ensiling. Apart from the 60th day of ensiling, the bacterial communities were clearly separated among the treated groups, and the bacterial communities of LP149 treatment were clearly separated from the other groups. Moreover, bacterial communities were clearly separated in silages stored at 30°C and those stored at 45°C. These suggest that temperature and additives are important factors affecting the bacterial communities of silage. Compared to additives, the temperature had a more profound effect on the bacterial community (Guan et al., 2020).

### Bacterial community composition during ensiling

The number of LAB is a key factor to determine the quality of silage. LAB can inhibit undesirable fermentation by rapidly lowering the pH (Chen et al., 2021). In this study, the number of LAB in all silages increased sharply compared with fresh hybrid elephant grass after 1 day of ensiling (see Supplementary Table). The LP149 treatment had a higher (*P* < 0.05) number of LAB than the other treatments throughout the fermentation process at 30°C.

**Figure 4:**
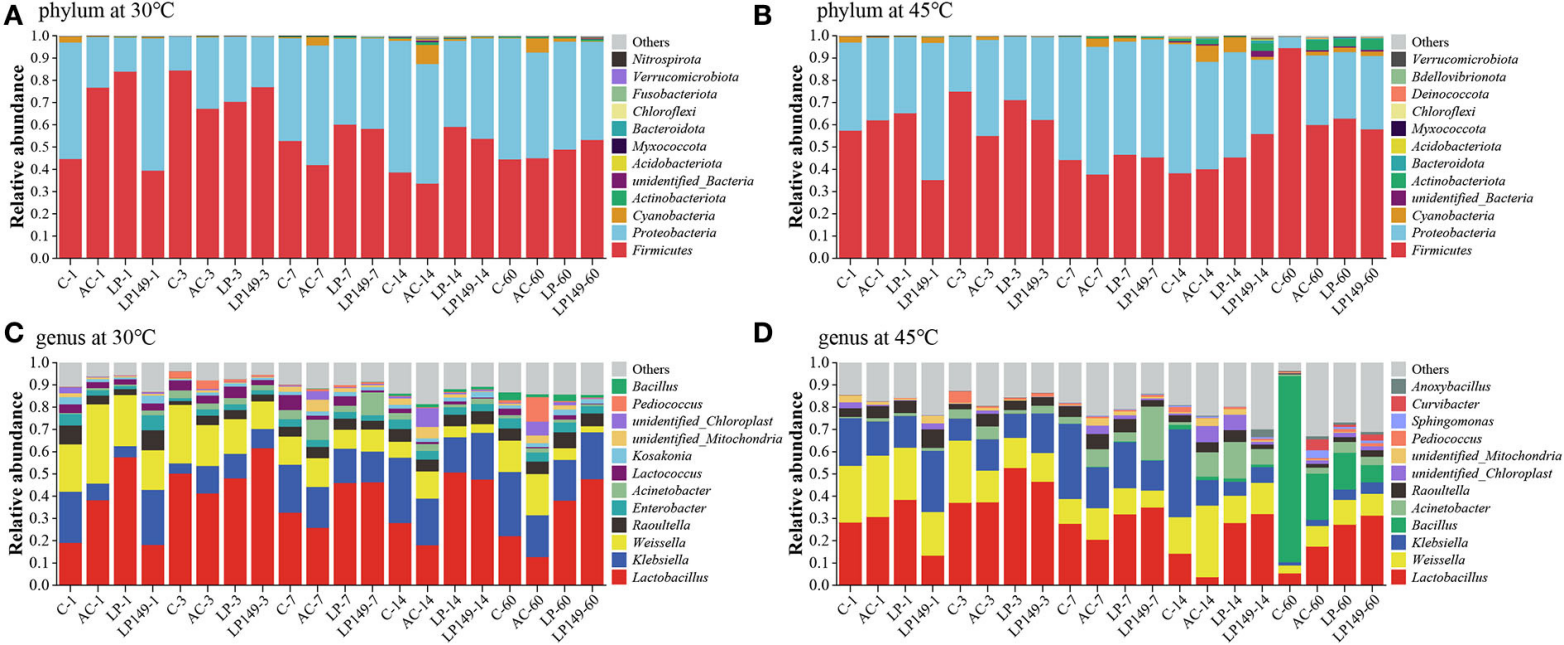
Relative abundance of bacterial community at phylum **(A,B)** and genus **(C,D)** level. C, control; AC, *Acremonium* cellulase; LP, commercial inoculant *Lactobacillus plantarum*; LP149, *Lactobacillus plantarum* 149. The number after the treatment zone represents the ensiling time: 1, 3, 7, 14, and 60 represent 1, 3, 7, 14, and 60 days of ensiling, respectively.

The bacterial community at the phylum and genus levels is presented in Figure 4. The main phyla in hybrid elephant grass silage were *Firmicutes* and *Proteobacteria*, and they are the most common phyla in silage. The main dominant bacteria in hybrid elephant grass silage were *Lactobacillus, Weissella*, and *Klebsiella*. In addition, the main bacteria involved in LA fermentation during ensiling were *Lactobacillus, Weissella*, and *Pediococcus*. The abundance of *Lactobacillus* in LP and LP149 treatments was higher than that in the control and AC treatments after 7 days of ensiling. This indicated that the addition of strains LP149 and LP could enhance the dominance of *Lactobacillus*. It is noteworthy that the AC treatment had a lower abundance of *Lactobacillus* than the control after 3 days of ensiling. This might be because some undesirable microorganisms have a growth advantage over epiphytic LAB under humid and hot environments (Pahlow et al., 2003), and AC treatment provides more fermentation substrate for them by degrading part of the lignocellulose into soluble sugars. *Weissella* might be an important bacterium for promoting LA fermentation in AC treatment. Generally, *Weisseria* will gradually decrease as prolonged fermentation time while acid-tolerant bacteria will increase (Ogunade et al., 2018). *Bacillus* has good acid tolerance and is found often in the late fermentation (Bai et al., 2020). After 60 days of ensiling at 45°C, *Bacillus* became one of the dominant bacteria. Particularly in control silage, *Bacillus* was the main dominant bacterium. It has been reported that *Bacillus* has the potential to improve the quality of silage (Bai et al., 2022), but the fermentation quality of the control silage was not good. Therefore, the role of *Bacillus* in silage in humid and hot areas needs further investigation.

*Klebsiella, Acinetobacter*, and *Raoultella* are the main undesirable bacteria in silage. These bacteria are usually considered undesirable microorganisms that compete with LAB for fermentation substrates (Du et al., 2022). Furthermore, *Klebsiella* and *Acinetobacter* can lead to reduced aerobic stability of silage (Lin et al., 2021; Muraro et al., 2021), whereas it was reported that *Klebsiella, Enterobacter*, and *Raoultella* are tolerant to low pH and can inhibit the pathogen (Gheibipour et al., 2022). Thus, it is necessary to carry out the in-depth study of bacteria normally considered undesirable during ensiling.

**Figure 5:**
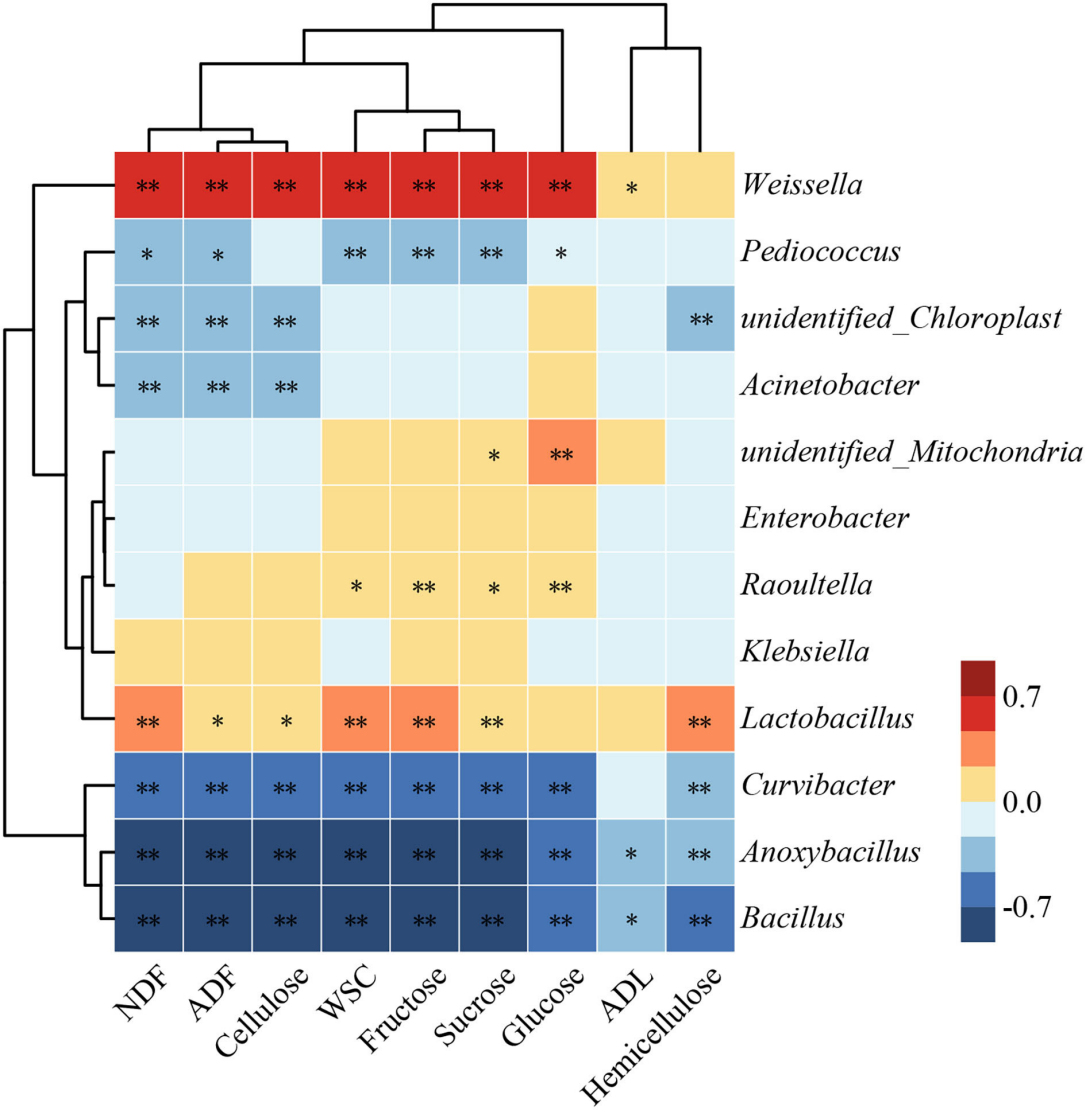
Heat map showing the correlations of dominant bacteria with lignocellulose and fermentable carbohydrates of hybrid elephant grass during ensiling. * and ** represent *P* < 0.05 and *P* < 0.01, respectively. NDF, neutral detergent fiber; ADF, acid detergent fiber; ADL, acid detergent lignin; WSC, water-soluble carbohydrates.

Bacterial systems have received much attention in the degradation of lignocellulose because of their outstanding functional diversity and adaptability (Georgiadou et al., 2021). The relationship among dominant bacteria with lignocellulose and fermentable carbohydrates of silage in humid and hot areas was investigated by Spearman's correlation analysis (Figure 5). The compositions of lignocellulose and fermentable carbohydrates were negatively (*P* < 0.05) correlated with *Bacillus, Anoxybacillus*, and *Curvibacter*. This demonstrated that microorganisms capable of promoting lignocellulose degradation may consume more fermentable carbohydrates during their action. Ahmed et al. (2018) reported that many members of *Bacillus* have ligninolytic and/or cellulolytic activities. In addition, *Acinetobacter* was negatively (*P* < 0.01) correlated with NDF, ADF, and cellulose. This is owing to that many species of *Acinetobacter* can promote the efficiency of carbohydrate catabolism and metabolism (Hošková et al., 2015). It is notable that the compositions of lignocellulose and fermentable carbohydrates were positively (*P* < 0.05) correlated with *Lactobacillus* and *Weissella*. This might be due to that these two bacteria rapidly lower the pH by involving in LA fermentation, which inhibits the consumption of soluble sugars by undesirable bacteria. The potential of LP149 to degrade lignocellulose may be due to that it directly or indirectly promoted the growth of bacteria with cellulolytic activity or as a result of its synergistic interactions with other bacteria.

## Conclusion

The AC and strain LP149 promoted the degradation of lignocellulose during ensiling of hybrid elephant grass. The addition of AC or LP149 altered the microbial communities' structure during ensiling. AC treatment provided more substrate for microbial fermentation, resulting in an increase in bacterial alpha diversity. LP149 treatment increased the *Lactobacillus* abundance and optimized the bacterial community compositions. In addition, AC and LP149 treatments increased lactic acid and acetic acid contents and decreased pH and protein breakdown, thereby improving the fermentation quality of hybrid elephant grass silage. Overall, AC and LP149 are promising additives that can improve the fermentation quality of high-moisture grass in humid and hot areas. It is possible that the combination inoculant with AC and strain LP149 may have better fermentation quality and is worth further study.

## Data availability statement

The datasets presented in this study can be found in online repositories. The names of the repository/repositories and accession number(s) can be found below: https://www.ncbi.nlm.nih.gov/, PRJNA894830.

## Author contributions

TZ, ZD, and YY designed the study. CC, YX, and HN performed the experiments. CC wrote the manuscript. XL, HG, YW, WY, YC, and YY revised the manuscript. All authors reviewed and approved the final manuscript.

## Funding

This study was supported by the National Natural Science Foundation of China (grant number 32001401) and Sichuan Science and Technology Department Programs (grant number 2021YFH0155).

## Conflict of interest

The authors declare that the research was conducted in the absence of any commercial or financial relationships that could be construed as a potential conflict of interest.

## Publisher's note

All claims expressed in this article are solely those of the authors and do not necessarily represent those of their affiliated organizations, or those of the publisher, the editors and the reviewers. Any product that may be evaluated in this article, or claim that may be made by its manufacturer, is not guaranteed or endorsed by the publisher.
