# Supplementary material for: Effects of Acremonium cellulase and heat-resistant lactic acid bacteria on lignocellulose degradation, fermentation quality, and microbial community structure of hybrid elephant grass silage in humid and hot areas
Source: Front Microbiol. 2022 Nov 21;13:1066753. doi: 10.3389/fmicb.2022.1066753 (PMC9719956; doi:10.3389/fmicb.2022.1066753)
Supplement: Supplementary file 1 [file Table_1.DOCX]

**Table 4**

Count of microorganisms in fresh hybrid elephant grass and ensiling process.

| Items | Fresh forage | Additives | 30℃ | | | | |  | 45℃ | | | | | SEM |
| --- | --- | --- | --- | --- | --- | --- | --- | --- | --- | --- | --- | --- | --- | --- |
|  |  |  | 1d | 3d | 7d | 14d | 60d |  | 1d | 3d | 7d | 14d | 60d |  |
| LAB  (log_10_ cfu/g of FM) | 2.03 | C | 6.21^d^ | 6.25^d^ | 6.41^b^ | 4.50^b^ | 2.03^e^ |  | 5.87^e^ | 6.00^e^ | 6.18^c^ | 2.68^f^ | 3.00^d^ | 0.227 |
|  |  | AC | 6.91^b^ | 6.73^b^ | 6.45^b^ | 4.03^c^ | 3.39^c^ |  | 6.85^bc^ | 5.92^e^ | 3.46^d^ | 3.16^e^ | ND |  |
|  |  | LP | 6.74^c^ | 6.60^c^ | 6.23^c^ | 3.30^d^ | 3.54^b^ |  | 5.98^e^ | 4.79^g^ | ND | ND | ND |  |
|  |  | LP149 | 7.69^a^ | 7.27^a^ | 7.01^a^ | 5.17^a^ | 5.89^a^ |  | 6.19^d^ | 5.01^f^ | ND | ND | ND |  |
| Coliform bacteria (log_10_ cfu/g of FM) | 4.00 | C | 3.39^a^ | ND | ND | ND | ND |  | ND | ND | 3.16^a^ | ND | ND | 0.087 |
|  |  | AC | 1.52^c^ | ND | ND | ND | ND |  | ND | ND | 2.17^b^ | ND | ND |  |
|  |  | LP | 1.09^d^ | ND | ND | ND | ND |  | ND | ND | 1.79^c^ | ND | ND |  |
|  |  | LP149 | 2.61^b^ | ND | ND | ND | ND |  | ND | ND | 2.06^b^ | ND | ND |  |
| Molds  (log_10_ cfu/g of FM) | 2.49 | C | ND | ND | ND | ND | ND |  | ND | ND | ND | ND | ND | 0.055 |
|  |  | AC | <1 | ND | ND | 1.87 | ND |  | ND | ND | 3.16 | ND | ND |  |
|  |  | LP | ND | ND | ND | ND | <1 |  | ND | ND | ND | ND | ND |  |
|  |  | LP149 | ND | <1 | <1 | ND | ND |  | ND | ND | ND | ND | ND |  |
| Yeasts  (log_10_ cfu/g of FM) | 1.75 | C | ND | ND | ND | ND | ND |  | ND | ND | ND | ND | ND | 0.103 |
|  |  | AC | ND | ND | 1.86^b^ | ND | 3.16^a^ |  | ND | ND | 5.16^a^ | ND | ND |  |
|  |  | LP | ND | ND | ND | 2.00 | 2.70^b^ |  | ND | ND | ND | ND | ND |  |
|  |  | LP149 | ND | <1 | ND | <1 | 2.67^b^ |  | ND | ND | ND | ND | ND |  |

^a^ Values with different lowercase letters show significant differences among treatments in the same ensiling day (*P* < 0.05); cfu, colony-forming unit log_10_ cfu /g of FM presents the total number of microbial communities per gram of sample in common logarithm values; FM, fresh matter; ND, not detected. SEM, standard error of means.

^b^ C, control; AC, *Acremonium* cellulase; LP, commercial inoculant *Lactobacillus* *plantarum*; LP149, *Lactobacillus* *plantarum* 149.
